# Psychological and social consequences among mothers suffering from perinatal loss: perspective from a low income country

**DOI:** 10.1186/1471-2458-11-451

**Published:** 2011-06-09

**Authors:** Kaniz Gausia, Allisyn C Moran, Mohammed Ali, David Ryder, Colleen Fisher, Marge Koblinsky

**Affiliations:** 1 International Centre for Diarrhoeal Diseases Research, Bangladesh (ICDDR,B).GPO Box 128, Dhaka 1000, Bangladesh; 2 Public Health, Edith Cowan University, 270 Joondalup Drive, WA 6027, Western Australia, Australia; 3 Combined Universities Centre for Rural Health, University of Western Australia, PO Box 109, Geraldton, WA 6531, Western Australia, Australia; 4 Department of International Health, Johns Hopkins School of Public Health, Wolfe St 8132, Baltimore, Maryland, USA; 5 Centre for International Health, Curtin Health Innovation Research Institute, Curtin University of Technology, WA 6845, Western Australia, Australia; 6 School of Population Health, University of Western Australia, Crawley-6009, Western Australia, Australia; 7 John Snow Inc., Rosslyn, VA 22209-3110, USA

**Keywords:** Perinatal death, postnatal depression, social consequences, rural women, Bangladesh

## Abstract

**Background:**

In developed countries, perinatal death is known to cause major emotional and social effects on mothers. However, little is known about these effects in low income countries which bear the brunt of perinatal mortality burden. This paper reports the impact of perinatal death on psychological status and social consequences among mothers in a rural area of Bangladesh.

**Methods:**

A total of 476 women including 122 women with perinatal deaths were assessed with the Edinburgh Postnatal Depression Scale (EPDS-B) at 6 weeks and 6 months postpartum, and followed up for negative social consequences at 6 months postpartum. Trained female interviewers carried out structured interviews at women's home.

**Results:**

Overall 43% (95% CI: 33.7-51.8%) of women with a perinatal loss at 6 weeks postpartum were depressed compared to 17% (95% CI: 13.7-21.9%) with healthy babies (p = < 0.001). Depression status were significantly associated with women reporting negative life changes such as worse relationships with their husband (adjusted OR = 3.89, 95% CI: 1.37-11.04) and feeling guilty (adjusted OR = 2.61, 95% CI: 1.22-5.63) following the results of their last pregnancy outcome after 6 months of childbirth.

**Conclusions:**

This study highlights the greatly increased vulnerability of women with perinatal death to experience negative psychological and social consequences. There is an urgent need to develop appropriate mental health care services for mothers with perinatal deaths in Bangladesh, including interventions to develop positive family support.

## Background

Perinatal mortality (stillbirth or death within the first 7 days of life) is widespread in low income countries^a^[1] and the burden of perinatal mortality falls disproportionately on low income populations including Bangladesh. Indeed, the World Health Organization estimates that the overall perinatal mortality rate in low income countries in 2000 was 61 per 1000 total births compared with 10 per 1000 in high income more developed countries [2]. Like all deaths, perinatal mortality can have a major emotional impact on members of the affected family, particularly the mother, with significant repercussions on the health and well-being of the family.

Studies have shown an increased incidence of depressive symptoms, guilt, prolonged grieving, and feelings of loss of control among parents after a perinatal loss [3-5]. Mothers who have lost their babies either by stillbirth or neonatal death are 7-9 times more likely to suffer from depression than women with a live baby [6]. As time elapses after bereavement, the degree of distress in parents can gradually decrease [7,8]. A 30 month follow up study on bereaved mothers noted a substantial reduction in the incidence of distress over the study period, from 21% at 2 months, 14% at 8 months to 10% at 30 months [8].

However, there is also evidence that some bereaved mothers suffer from long term psychological distress after such loss. For instance, a number of studies have documented that some bereaved women encounter long term consequences including depression in subsequent pregnancies and prolonged grief reactions and marital disharmony that lead to separation and divorce [9-11]. Beutel et al. (1995) followed up women up to 1 year after miscarriage and found that bereaved women suffered longer lasting negative psychological, physical and social changes following initial depression [10]. Depressed mothers often felt guilt and shame at losing their unborn babies. Such women were often profoundly sad, cried, and yearned for their lost child. Although baseline depression status was not associated with partnership breakdown, poor perceived support from partner at times of loss predicted subsequent partnership breakdown, as seen in a Swedish study that followed a cohort of women for 7 years after perinatal loss [11]. The risk of partnership breakdown was 4 times higher among women with stillbirths compared to the women who had live babies.

Regarding social consequences, data shows that grieving parents also have to contend with a wide range of negative social effects following a perinatal death, such as isolation from friends, extended family members, and others in their social networks leaving them more emotionally vulnerable [12]. While these psychological and social consequences have been quite well studied and identified in developed countries, there is considerably less information about these from developing countries.

The limited data available from developing countries shows that a significant proportion of women experience depression after perinatal loss. Recent cross-sectional studies in Africa (n = 108) and in Malaysia (n = 62) indicate that around half of all women with perinatal death have high levels of depressive symptoms in the postnatal period [13,14]. Factors significantly associated with perinatal losses were poor support from husband, pregnancy complications and previous history of perinatal loss in the African study, whereas previous history of perinatal loss was not associated with depression status among Malaysian women. Bereaved Malaysian women who had no support from their friends experienced significantly higher depressive symptoms. However, the results of these studies should be interpreted cautiously because of the study design, selection of the study participants, small sample size and hospital based data. Therefore, there is a need to explore in greater depth the community based data and from larger samples to understand the implications of perinatal death on affected women.

In Bangladesh, the perinatal mortality rate is 55/1000 pregnancies, most of which occur in the rural areas [15]. While many medical and socio-cultural reasons have been identified for this loss [16,17], there is very little known about the psychological and social consequences for the women who have lost their babies. An earlier study we carried out in rural Bangladesh indicated that perinatal death was one of the strongest predictors of postnatal depression [18]. However, the rate of depression and social sequel among women with perinatal loss are largely unknown.

The purpose of this prospective community-based study was to estimate the magnitude of psychological and social consequences of childbirth resulting in perinatal death in rural Bangladeshi women. We hypothesized that women who had experienced perinatal loss (e.g. stillbirth, early neonatal death) would be more likely to have depression and will encounter impaired social function compared to those with normal deliveries with no complications. We hope that the study findings can be used to inform discussions around programme interventions and policies to address maternal psychosocial health and welfare following perinatal death.

## Methods

### Study site

The study was conducted in the rural sub-district of Matlab, 55 kms from the capital Dhaka, Bangladesh. People in Matlab earn their livelihood mainly from agriculture and fishing. Although overall economic conditions and literacy rates have improved in Bangladesh over the past ten years, a 2005 socio-economic survey in Matlab found that 37% of women have no education, with only 12% having completed 10 years or more of schooling [19]. Few women work outside the home, with the majority engaged in household work and child rearing.

A Maternal and Child Health (MCH) programme in Matlab was initiated in 1987 by the International Centre for Diarrhoeal Disease Research, Bangladesh (ICDDR, B), covering a population of 110,000 in *four geographic blocks (Block A, B, C and D)*. The programme maintains four MCH clinics, one in each block which provide free antenatal care, basic essential obstetric, postnatal care, family planning and child health services. The programme also maintains health and demographic surveillance (HDSS) records on vital events such as births, deaths, marriages, divorces, and in-and out-migration through bi-monthly home visits by community health research workers (CHRWs) [20]. The information is maintained in a computerized database which is updated on a regular basis.

### Recruitment of study subjects

**Figure 1:**
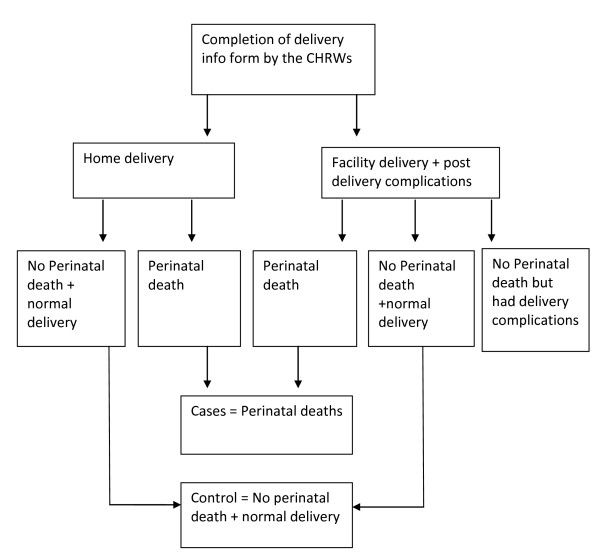
**Process of sample selection**.

This prospective community-based study is part of a larger 'Burden of maternal ill health study' conducted in the MCH project area to estimate physical, economic, social, and psychosocial consequences of childbirth among women with normal births, obstetric complications, and perinatal deaths. During the study period (January 2007 to March 2008), CHRWs documented the birth location and outcome (e.g: stillbirth, live birth) among women residing in the project area with a recent delivery on a fortnightly basis. Among women with facility births in the project area or in close proximity (30 km radius of project area), two study physicians reviewed medical records using a structured form to assess obstetric complications. The list was continuously updated as soon as CHRWs received information on delivery and after medical records were reviewed by the project physicians. In this way, all women with a perinatal death and a random sample of women with normal births (excluding severe obstetric complications) were eligible for inclusion in the study (Figure 1). Cases of perinatal death were defined as still births (after 28 weeks of gestation) and/or death of a live born baby within seven days of birth. For each perinatal death, two unmatched controls with normal deliveries were selected. Normal deliveries were defined as singleton, cephalic and full term (> 37 weeks of pregnancy) normal vaginal delivery either at home or at a health facility and whose medical records revealed no complications during delivery or the immediate postpartum period.

In the study, 122 women with perinatal death and 354 women in the comparison group with no perinatal death were assessed at 6 weeks postpartum and then were followed up at 6 months after childbirth.

### Data collection and Instruments used

All interviews were carried out by three trained local female interviewers at the womens' homes at 6 weeks and 6 months postpartum. The interviewers received classroom training on data collection techniques, childbirth related issues, and tips on successful applications of mental health assessment questionnaire. Also, role play techniques were used to teach interviewers how to deal with emotional issues while collecting information from bereaved participants. Confidentiality and privacy were maintained and formal consent sought before proceeding with interviews. If women were not found at home at the time of the scheduled interviews, follow-up visits were carried out for an additional 2 weeks to see whether they had become available for the interview. At the first interview at 6 weeks postpartum, a brief socio-demographic questionnaire was administered followed by mental health assessment. Socio-demographic data was also supplemented by information on women's economic status (assets) and parity maintained by the HDSS system of ICDDRB.

The 6-month postpartum follow-up visit consisted of 2 closely spaced home visits, one to interview the women regarding their mental health status and the other to elicit information on social consequences.

### Assessment

Women's mental health was assessed with a locally validated Bangla version of the Edinburgh Postnatal Depression Scale (EPDS-B) [21]. The EPDS is an internationally accepted screening instrument developed by Cox and colleagues in 1987 and has been widely used both in developed and developing countries [22]. The scale consists of 10 items or statements each with four responses about the respondent's emotional wellbeing during the preceding 7 days. Based on responses an individual can achieve a minimum total score of 0 to a maximum of 30. An earlier study in Bangladesh with a locally validated EPDS (EPDS-B) found that a total cut-off score of 10 was optimum in identifying possible depression cases in the community [21].

The questionnaire on social consequences assessed the impact of the childbirth on the women's social context, including perceived health status, ability to perform daily activities including household chores, perceived life changes, relationships with family and neighbors, ability to care for her children, and return to paid work. This pre-coded questionnaire was developed based on a similar instrument used in a study in Burkina Faso, West Africa (personal communication with Author AM). It was adapted to the Bangladeshi context and translated into Bengali, extensively pre-tested and revised before applying to study women. For this analysis, questions included items related to perceived life changes since last birth, their feelings and relationships with their marital family, their community, and husbands. Life changes were assessed through asking "Do you think your life has changed positively, negatively, or not at all since your last delivery?" Relationships were examined with "Compared to what it was before your pregnancy, is your current relationship with your marital family generally better, worse, or more or less the same?" A similar pattern of questions was followed to assess relationships with community and husbands.

### Data analysis

SPSS windows version 17 was used to derive descriptive statistics comparing women with perinatal death with those who had no perinatal death. Univariate associations between perinatal death, depression, and social circumstances were tested using Pearson's chi-square or Fisher's exact test. Associations between psychological distress (derived from total EPDS-B scores) and social consequences at 6 months after childbirth were investigated using logistic regression analysis adjusting for possible confounders. With limited knowledge on temporal relationships of cause and effect, it is not certain whether a specific parameter was a cause or effect of psychological distress after childbirth. For this reason variables were only included in the regression model if they were a main exposure under investigation (e.g. perinatal death) or a confounder such as age, education, area of residence and socio-economic status. Variables that were found significantly (p < 0.05) associated with perinatal death were also included in the logistic regression analysis.

### Ethical considerations

Ethical clearance was obtained from the research and ethical review committees of ICDDR, B. Informed consent was obtained before recruitment. Appropriate medical and psychological services were provided to participants if they were thought to need such services during the study duration.

## Results

### Obstetric history

A total of 476 women were interviewed at six weeks following childbirth, of which 122 had a perinatal death. Two thirds (67%) of the perinatal deaths were still births, and the remaining third early neonatal (within 7 days of birth) deaths. The majority (69%, n = 84) of the perinatal deaths occurred at a health facility. No medical records were available for 32 perinatal deaths. A little over one third (37%, n = 31) of women with perinatal deaths had normal vaginal delivery, three had a Caesarean section and 21% (n = 18) had obstetric complications (e.g. prolong labour, malpresentations, breech delivery, eclampsia). Data presented here are for women who had completed both 6 weeks and 6 months assessments.

Table 1 shows selected socio-demographic characteristics of study women with and without perinatal deaths. Background characteristics, for instance, age, education level and income status did not differ significantly between women experiencing perinatal loss and those not experiencing such loss. However, there was a significant association between perinatal death and lower parity (0, 1, 2 births). A substantially higher proportion of women in the perinatal death group were living with their in-laws compared to the women with no perinatal deaths (51% Vs 39%, p = 0.02).

**Table 1:** Socio-demographic characteristics of the study population

	Perinatal death Chi-square		Chi-square (p value)
		
	Yes (n = 122)	No (n = 354)	
Background characteristics			
**Maternal age**			1.364 (0.850)
Less than 20 yrs	20 (16.4)	50 (14.1)	
20-24 yrs	38 (31.1)	107 (30.2)	
25-29 yrs	35 (28.7)	102 (28.8)	
30-34 yrs	18 (14.8)	67 (18.9)	
35 + yrs	11 (9.0)	28 (7.9)	
**Education**			3.561(0.313)
No education	25 (20.5)	62 (17.5)	
Primary (1-5 yrs)	32 (26.2)	113 (31.9)	
Secondary (6-10 yrs)	58 (47.5)	169 (47.7)	
Above secondary	7 (5.7)	10 (2.8)	
**Residential area**			5.852 (0.119)
Block A	42 (34.4)	92 (26.0)	
Block B	33 (27.0)	109 (30.8)	
Block C	29 (23.8)	73 (20.6)	
Block D	18 (14.8)	80 (22.6)	
**Family structure**			4.965 (0.02)
Nuclear family	60 (49.2)	215 (60.7)	
Joint family	62 (50.8)	139 (39.3)	
**Parity**			34.96 (0.000)
1-2	59 (48.4)	83 (23.4)	
More than 2	46 (37.7)	240 (67.8)	
Unknown	17 (13.9)	31 (8.8)	
**Economic status (Asset quintile)**			5.162 (0.396)
Poorest	21 (17.2)	69 (19.5)	
Less poor	22 (18.0)	60 (16.9)	
Middle	20 (16.4)	71 (20.1)	
Richer	17 (13.9)	66 (18.6)	
Richest	31 (25.4)	64 (18.1)	
Unknown	11 (9.0)	24 (6.8)	

### Mental health and social factors

Overall, at 6 weeks postpartum, one quarter (23.7%, n = 113) of all women had an EPDS-B score of 10 or above, indicating possible postpartum depression. However, the rate of depression decreased over the postpartum period and just over a tenth (12%, n = 57) of women had an EPDS-B score of 10 or above at 6 months postpartum. Table 2 shows the distribution of EPDS-B items scores with depression status among women at 6 weeks and 6 months postpartum. Each item was independently associated with depression status at both follow-up periods (Table 2).

**Table 2:** Depression symptoms following childbirth among study women

	6 weeks postpartum				6 months postpartum			
**EPDS-B items**	**Depressed** **n = 113 (%)**	**Non-depressed** **n = 363 (%)**	**Odds** **ratio**	**95% CI**	**Depressed** **n = 57 (%)**	**Non-Depressed** **n = 419 (%)**	**Odds ratio**	**95% CI**

**Not able to laugh and see funny things**	103 (41.2)	147 (58.8)	9.31	5.00-17.37	54 (31%)	120 (69%)	31.24	9.92-98.42
**Did not look forward with enjoyment**	90 (42.7)	121 (57.3)	4.91	3.23-7.49	47 (35.6)	85 (64.4)	12.25	6.38-23.52
**Had feelings of guilt**	94 (38.1)	153 (61.9)	4.58	2.90-7.26	51 (24.8)	155 (75.2)	11.14	4.88-25.45
**Was anxious**	73 (51.4)	69 (48.6)	4.29	3.08-5.98	37 (45.7)	44 (54.3)	9.02	5.54-14.71
**Was panicky**	62 (61.4)	39 (38.6)	4.51	3.35-6.08	27 (50.0)	27 (50.0)	7.03	4.55-10.88
**Was overwhelmed**	87 (43.9)	111 (56.1)	4.70	3.15-7.00	45 (21.4)	165 (78.6)	4.75	2.58-8.75
**Had a problem sleeping**	91 (66.9)	45 (33.1)	10.34	6.79-15.76	42 (40.4)	62 (59.6)	10.02	5.79-17.32
**Had feelings of helplessness**	102 (54.5)	85 (45.5)	14.33	7.91-25.96	45 (21.4)	165 (78.6)	4.75	2.58-8.75
**Cried**	95 (55.2)	77 (44.8)	9.33	5.84-14.89	42 (40.4)	62 (59.6)	10.01	5.79-17.32
**Had thoughts of self harm**	30 (81.1)	7 (18.9)	4.29	3.35-5.50	50 (36.2)	88 (63.8)	17.50	8.14-37.63

Women who were depressed at 6 weeks postpartum were 9 times more likely to report "crying", being unable to 'laugh and see the funny side of things', and experiencing sleep problems over the past week compared to participants who were non-depressed. The most remarkable symptoms were feelings of helplessness among depressed women. They were 14 times at higher risk of reporting 'helplessness' compared to the women who were non-depressed. In addition, depressed women were 4-5 times more likely to report self harm ideation, and were more likely to report being 'anxious' and 'panicky' compared with their non-depressed counterparts. A similar pattern of depression symptoms was reported among depressed women at the 6 months assessment. Depressed women at 6 months postpartum were 30 times more likely to report that they were 'not able to laugh and see the funny side of things'.

Study women with a perinatal death were significantly more likely to be depressed at 6 weeks postpartum than women who did not have a perinatal death (Table 3). After a perinatal loss, 43% (95% CI: 33.7-51.8%) of women were found depressed at 6 weeks postpartum compared to 17% (95% CI: 13.7-21.9%) with healthy babies at 6 weeks postpartum (p = < 0.001). However, at 6 months after childbirth, there was no statistically significant difference (p = 0.65) in the prevalence of depression between women with perinatal death and women who had not experienced perinatal death. *Further analysis was carried out to answer the question: Does depression persist among women suffering perinatal loss?*

**Table 3:** Mental health and Social Consequences among study women in Matlab, Bangladesh

	Perinatal death		Chi-square test
		
	Yes (n = 122)	No (n = 354)	
**Psychological consequences**	**(%)**	**(%)**	**(p value)**

Depression at 6 weeks	52 (42.6)	61 (17.2)	32.311(0.000)
Depression at 6 months	16 (13.1)	41 (11.6)	0.202 (0.653)

**Social consequences**			

Negative life change	32 (26.2)	34 (9.6)	20.997 (0.000)
Worse relationship with marital family	17 (13.9)	18 (5.1)	10.431(0.001)
Worse relationship with community	5 (4.1)	7 (2.0)	1.661 (0.197)
Worse relationship with husband	12 (9.8)	16 (4.5)	4.632 (0.031)
Not valued in marital family	79 (64.8)	188 (53.1)	4.997 (0.025)
Felt guilty as a result of last pregnancy	39 (32.0)	34 (9.6)	33.24 (0.000)

**Figure 2:**
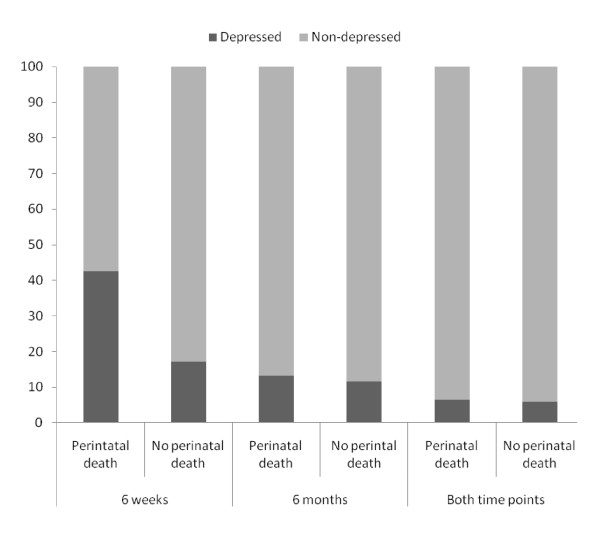
**Psychological status of mothers**.

Overall, a small proportion of women with perinatal death persistently suffered from depression. Of the 52 (43%) women with perinatal deaths who were depressed at 6 weeks assessment, 44 recovered from their depression status. At 6 months assessment, only 16 women with perinatal death (13.1%) were found depressed including 8 women (6.6%) who were persistently depressed from their six week assessment (Figure 2). On the other hand, around 6% of women with no perinatal deaths also suffered with persistent depression. The magnitude of persistent depression was statistically non-significant between women with and without perinatal deaths (Figure 2).

The univariate analysis shows that study women with a perinatal death were more likely to report negative social consequences compared to women without a perinatal death (Table 3). Women who experienced perinatal death reported significantly more negative life changes (p = < 0.001) than their counterparts with live babies.

Women had a higher frequency of reporting worsening of relationships with their husbands (p = 0.03) and with marital family members (p = 0.001) following the perinatal death than women without a perinatal death. These women also reported being significantly less valued (p = 0.03) by their marital family since the perinatal death, compared to women with live babies (Table 3). A substantial proportion of women with perinatal death reported feelings of guilt following the perinatal death compared to women with live babies (32% Vs 10%, p = < 0.001).

Table 4 presents the effects of perinatal deaths and reported social consequences on psychological distress after six months of childbirth along with adjusted odds ratios and significance levels. Although statistically non-significant, perinatal death was marginally associated with depression status at 6 months follow up. However, women who were depressed at 6 weeks postpartum remained significantly depressed (adjusted OR = 3.65; 95% CI: 1.85-7.21) at follow up even after controlling for age, education, family structure, residential area, and economic status. Similarly, women who felt guilty (95% CI: 1.22-5.63) about their results of last pregnancy outcome continued to be significantly depressed (adjusted OR = 2.61) at 6 months postpartum. Report of poor relationship with the husband (95% CI: 1.37-11.04) was independently associated with depression status (adjusted OR = 3.89) at 6 months postpartum.

**Table 4:** Effects of perinatal deaths and social consequences on psychological distress after six months of childbirth

Exposure^a^	Adjusted odds ratios	Wald	95% confidence Intervals	Significance level *P *value
**Perinatal death**	0.45	3.59	0.20-1.03	0.06
**Depression at 6 weeks postpartum**	3.65	13.91	1.85-7.21	< 0.001
**Negative life change**	1.29	0.34	0.55-2.99	0.56
**Worse relationship with marital family**	1.93	1.58	0.69-5.42	0.21
**Worse relationship with community**	0.62	0.29	0.11-3.53	0.59
**Worse relationship with husband**	3.89	6.50	1.37-11.04	0.01
**Not valued in marital family**	1.42	0.99	0.71-2.84	0.32
**Felt guilty as a result of last pregnancy**	2.61	6.04	1.22-5.63	0.01

a Adjusted for age, education, family structure, residential areas and asset quintiles.

## Discussion

This is one of the few community based prospective studies from a low-income Asian country with high perinatal mortality that explores the impact of perinatal deaths on women's mental health and social life. The results indicate that such women experience increased rates of depression at 6 weeks postpartum and suffer from negative social consequences that persist 6 months after the perinatal death.

This study found significantly higher levels of depressive symptoms in women with perinatal loss compared to non-bereaved mothers at 6 weeks postpartum. This finding is consistent with reports from other countries [14,23]. An important point to consider is whether the higher rates of depression among bereaved mothers can be attributed to the death of the baby, as rates of depression prior to delivery were not assessed. However, this study did demonstrate that women with perinatal deaths experienced depression at rates 3 times higher in the early (6 weeks) postpartum period than those who had not experienced such loss.

Depression among bereaved mothers declined markedly at 6 month postpartum, indicating recovery with time. Other studies have also found such a reduction in depression rates among bereaved mothers in the postpartum follow up period [3,24,25]. However, a clear understanding of the underlying mechanisms for such resolution of depression symptoms among bereaved mothers is yet to emerge. Further investigation should be carried out to see whether women who were depressed at six weeks and reported short term (six weeks) significant negative social consequences would have spontaneous social adjustment or have the same negative social consequences for long term at 12 months.

Our study also found a disturbingly high rate of adverse changes in the social circumstances of mothers experiencing perinatal loss, with deteriorating relationships and support from members of the marital family including their husbands. Bereaved mothers also felt guilty because of the outcome of their last pregnancy. These adverse circumstances may well have been under-reported, as women in Asian societies rarely complain about their relationships with their husband or other marital family members, as traditional customs frown upon married women talking against their marital family members or their husband [26,27].

This study results indicate the great need to address the psychological needs of women experiencing perinatal deaths in countries like Bangladesh which experience high rates of perinatal mortality. This need becomes even more pressing when one considers that these mothers are also often less likely to seek postnatal care for themselves [28,29]. While professional mental health support and help are widely available for bereaved mothers in developed countries, delivering such services in Bangladesh will be a challenging task, given that public mental health services do not extend to the sub-district level. There is only one psychiatrist for every two million people in the country and no mental health disorder is covered by social insurance [30]. A special approach using community health workers with training in culturally appropriate primary mental health care may be the answer [31].

### Strengths and limitations

This was a community based study and the consequences were recorded prospectively so there is less chance of recall bias in reporting social circumstances by study woman. The use of validated local version of internationally accepted EPDS-B scale has given the opportunity to compare the estimated magnitude of depression cross culturally and internationally. In addition, a comparatively larger sample size and a good sized comparison group contributed to achieving robust findings on perinatal death and its social and mental consequences. The scope of application of longitudinal data analysis approach has been missed because of the unavailability of repeated measures of social data at base line (6 weeks assessment). Asking women about their social circumstances at 6 weeks after childbirth was considered to be too early in their mourning period, as it could have influenced their responses.

## Conclusions

In resource-constrained settings where public mental health services are not well developed, the importance of support from family members cannot be overemphasized. Family support is the only form of social support that has been proven to have considerable benefit in reducing maternal anxiety and depression following perinatal loss [32]. As such, there is an urgent need to develop interventions that can harness the efforts of family members, including extended and marital family, towards providing positive, not negative, support for bereaved mothers.

## Competing interests

The authors declare that they have no competing interests.

## Authors' contributions

KG, MK and AM participated in the planning and conception of the research questions and the study design. KG guided the psychological and AM guided the social component of the study and analysed data jointly. MA, DR and CF provided help with data analysis and editing of the manuscript. All authors participated in interpreting the data and critically revised the manuscript. All authors read and approved the revised manuscript.

## Note

^a ^According to the World Bank economic indicator countries around the globe are classified based on their Gross National Income (GNI). Country with GNI per capita US$ 995 or less is classified as low income countries. The GNI per capita is US$ 580 for people of Bangladesh. http://data.worldbank.org/about/country-classifications

## Pre-publication history

The pre-publication history for this paper can be accessed here:

http://www.biomedcentral.com/1471-2458/11/451/prepub
